# Temperature and Morphology Affect the Performance and Cost of Flight in Spruce Budworm Females

**DOI:** 10.1002/ece3.72529

**Published:** 2025-11-29

**Authors:** Lucie Royer, Jacques Régnière

**Affiliations:** ^1^ Natural Resources Canada, Canadian Forest Service, Laurentian Forestry Centre Quebec Canada

**Keywords:** distance, duration, flight propensity, physiology, speed, wingbeat

## Abstract

Dispersal is a key process in the spatial and temporal dynamics of insect populations. Dispersal depends on the flight performance of individual insects, which is influenced by their environment, morphology, and physiological state. Temperature affects flight performance and costs through its effect on the metabolism of ectotherms. It can also induce developmental changes in flight‐related traits that influence flight biomechanics and insect size, affecting the resources available for this activity. We thus need to understand how temperature during flight modulates flight performance and costs, but also how morphology affects them. Using flight mills, models were developed to describe how flight performance and costs of individual spruce budworm females varied over a range of temperatures (10°C–35°C). Variations of barometric pressure and morphological traits were also incorporated in these models. Flight propensity dropped below 20°C, and when female mass increased relative to wing area, suggesting that there is a wing load beyond which flight probability decreases. Speed, duration, and distance of flight decreased as temperature deviated from 23°C, while wingbeat frequency increased consistently with temperature. Females with long and broad wings had greater flight endurance. Mass loss and use of water and fuel (lipids and carbohydrates) increased with temperature, while the remaining lipids were not affected. As a result, female budworms allocated a daily energy budget to flight, which was proportional to their mass. Large females also benefited from an energy saving due to their mass during flight. Water loss was proportional to female mass but decreased with distance traveled, reaching hydric quasi‐homoeostasis at distances greater than 10 km. Our approach reveals the underlying mechanisms of flight and highlights the factors that influence the landing process after dispersal in the spruce budworm. The relationships presented in this study can help develop more realistic models of dispersal for this boreal forest pest.

## Graphical abstract

Forewing length and wing surface are the key morphological traits to explain the biomechanics of the flight performance in spruce budworm. Large budworm females benefit from more energy reserves that can be allocated daily to flight and a relative energy saving during flight due to their mass. Together, wing morphology and the rate at which the daily energy budget runs out with temperature influence the flight endurance of budworm females and determine where they land after dispersal.

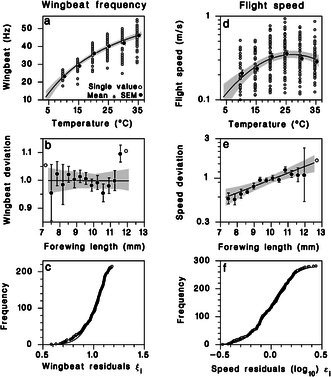

## 1 Introduction

Dispersal influences the spatial distribution, gene flow, and population dynamics of species, as well as the dynamics and synchrony of metapopulations (Bowler and Benton 2005; Ronce 2007). Most studies on the dispersal of Lepidoptera are done at the population level (Holland et al. 2006; Holyoak et al. 2008). However, dispersal is the result of self‐propelled or wind‐borne flight by many individual insects (Hu et al. 2016; Satterfield et al. 2020). To understand the spatial movement of a species in a changing climate, we thus need to know not only the ability of each insect to fly, but also the factors that affect its flight performance (flight propensity, propulsion and endurance), such as its physiological state, morphology and environment (Dudley 2000; Goossens et al. 2020; Nathan et al. 2008).

In Lepidoptera, resources acquired at the larval stage are allocated to the different parts of the adult body during metamorphosis and determine the physiological state and morphology of the adult at emergence. In species that do not feed as adults, the resources they can invest in reproduction, maintenance, and flight thus depend entirely on the physiological state of the adults at emergence (Boggs 2009). As the mass of an insect is often a substitute for its physiological state, variation in adult mass after flight can be used to assess the amount of resources invested in this activity or its cost (Goossens et al. 2020). Lipids are the principal fuel for sustained flight in Lepidoptera, although some use carbohydrates at the beginning of a flight bout (Beenakkers et al. 1984). The performance and cost of flight also depend on biomechanical efficiency, which is related to morphological traits of the individual. These traits include wing length, wing area, wing aspect ratio, wing load, and relative thorax mass (Dudley 2000; Viljur et al. 2019 and references therein). These traits may be affected by temperature during insect development (Gibbs et al. 2011; Günter et al. 2020; Minter et al. 2024). In the context of climate change, this developmental plasticity can have an impact on the biomechanics of flight. In addition, flight performance and cost can be modulated by the environment. Among environmental factors, temperature is probably the most important as it affects insect metabolism directly (Mattila 2015; Niitepõld 2010). The performance and cost of flight usually increase with metabolism, but flight propensity, propulsion, and endurance can rapidly drop as thermal conditions move away from an optimum or approach the tolerance thresholds of the insect (Berwaerts and Van Dyck 2004; Mattila 2015; Sinclair et al. 2016). The development of thermal performance functions for the various flight components helps understand and predict insect movement in response to climate change (Hillaert et al. 2015; Sinclair et al. 2016). We need to understand not only how temperature shapes the performance and cost of flight, but also how physiological and morphological traits affect them in interaction. However, to our knowledge, there is no model of performance and cost of flight that integrates both temperature and morphological traits. In this study, we filled this gap by using a highly mobile insect as a model: the spruce budworm.

The spruce budworm, *Choristoneura fumiferana* (Clem.), is a native univoltine lepidopteran that defoliates large areas of coniferous forest in North America (MacLean 2016). Outbreaks of this insect occur every 30 to 43 years, last an average of 10 years and can cover several million hectares (MacLean 2016; Royama 1984), often leading to extensive host tree mortality, especially balsam fir, *Abies balsamea* (Linnaeus) Miller (Pinaceae), and white spruce, *Picea glauca* (Moench) Voss (Pinaceae) (MacLean 2016). Spruce budworm moths are not known to feed (Harvey 1977). It is thus likely that they depend only on resources acquired during the larval phase for reproduction, maintenance, and flight. Historically, daily mean temperatures during the flight of this species can range from 10.7°C to 21.3°C in the area where the current outbreak in Quebec began, but extreme temperatures of 3.0°C and 32.0°C have been recorded (Environment Canada 2025). Moth dispersal is a central process in the population dynamics of this insect (Greenbank 1957; Johns et al. 2019; Larroque et al. 2020; Régnière and Nealis 2019; Royama 1984). Over the years, several events of wind‐borne mass flights of budworm moths have been documented (Boulanger et al. 2017; Dobesberger et al. 1983; Greenbank 1957; Greenbank et al. 1980; Rhainds et al. 2022). Dispersing moths fly vertically up to an altitude of 400–800 m, where winds can carry them hundreds of kilometers from their starting point before settling (Boulanger et al. 2017). A mechanistic model of budworm moth dispersal has been developed (Garcia et al. 2022, 2024; Régnière, Cooke, et al. 2019; Régnière, Delisle, et al. 2019), with at its core a relationship between wingbeat frequency and temperature that was deduced from field observations (Régnière, Delisle, et al. 2019). Many factors that determine whether the moths leave and where they land following dispersal remain poorly understood. Most of the laboratory work on budworm flight has focused on flight propensity based on time of day, sex, size, mating status, age, and temperature (Edwards 1962; Sanders and Lucuik 1975; Sanders et al. 1978). However, flight propensity is a binary variable, indicating whether the insect flies or not, and does not give any indication of flight duration or distance covered. Flight quantified in this way may simply be related to routine behavior within the natal site, unrelated to dispersal by self‐propelled or wind‐borne flights. Some components of flight performance, such as speed, 1‐h flight distance, and maximum sustained flight duration, were evaluated in only one laboratory study to determine the effect of food limitation at the last larval stage (Van Hezewijk et al. 2018). More empirical data on the flight performance of individuals are therefore needed.

In this study, we used flight mills to quantify the flight performance and costs of virgin budworm females over a period covering their entire daily activity under controlled conditions. Within a temperature range of 10°C–35°C, we developed models of thermal performance for propensity, wingbeat frequency, speed, duration, and distance that incorporate individual variability. In each model, we assessed the contribution of several factors: temperature, barometric pressure, and morphological traits known to affect flight biomechanics. The most parsimonious models were selected according to their Akaike Information Criterion (AIC) of information theory (Angilletta 2006). To estimate the cost of flight, we measured the mass of females before and after flight and determined the lipids remaining after flight. From these data, we developed models to predict the mass loss of female individuals, the amount of water and fuel (lipids and carbohydrates) used during flight, and the amount of lipids remaining after flight as functions of temperature, and to highlight the morphological traits that affect the energy efficiency of flight.

## 2 Materials and Methods

### 2.1 Experimental Methods

#### 2.1.1 Source of the Insects and Their Rearing

Second instar larvae of the spruce budworm (Glfc: IPQL: Cfum15; Roe et al. 2018) and an artificial diet based on wheat germ (McMorran 1965) were obtained from the Canadian Forest Service Insect Production and Quarantine Laboratories in Sault Ste. Marie, Ontario, Canada. Groups of 10 larvae were placed on diet in 30 mL cups (model P100, Solo Cup Company, Toronto, ON, Canada), and reared at 25.0°C ± 0.5°C, 50% ± 5% RH, under a photoperiod of 16 L: 8D. These constant developmental conditions are optimal for this species (Régnière et al. 2012) and allowed us to more easily predict adult emergence, although the main daily temperature during larval development in the boreal forest can vary from 2.0°C–12.3°C at the end of the second instar diapause in spring to 10.7°C–21.3°C at the pupal stage in mid‐summer (Environment Canada 2025). At pupation, females were placed individually in 30 mL cups, and moth emergence was checked daily. The newly emerged females were isolated in plastic bags (Poly 2 lb., Les emballages L. Boucher, Québec, QC, Canada) filled with air, and kept under the same conditions as the rearing. The next day, females that did not lay eggs were weighed to the nearest 0.01 mg (model Cubis II MSE 225 s‐100‐DU, Sartorius Canada Inc., Oakville, ON, Canada) to determine their initial mass, which also corresponded to the counterweight required for the flight mill. As a result, virgin spruce budworm females were tested on their second day of life, when they are most active, at a level equivalent to that of egg‐laying, mated females on their third day (Sanders and Lucuik 1975).

#### 2.1.2 Flight Performance

Each female was cooled on an ice pack (Ice‐Pak IP‐050; Cryopak, Montréal, QC, Canada) for a few minutes to calm it down. The dorsal hairs of the thorax were removed, and an insect pin was glued to it with low‐temperature hot melt glue. The pin attached to the moth was then inserted into one arm of a rotary flight mill (model 15‐FMASM, Crist Instrument Company, Hagerstown, MD, USA), and a counterweight equivalent to the female initial mass ±5 mg on average was added at the other end of the arm. All females were installed before 13:00 in the growth chamber, where flight tests were performed at ambient temperatures of 10°C, 15°C, 20°C, 25°C, 30°C, or 35°C (all ±1°C) and 54% ± 5% RH. Females then had at least 30 min to acclimate to the growth chamber conditions before recording high‐speed videos.

To determine the effect of temperature on wingbeat frequency, females that were not flying spontaneously were stimulated by lightly touching one wing with a soft bristle brush. Females were considered unwilling to fly after three stimuli without reaction or if they did not make more than one complete rotation on the flight mill. Videos of the responsive females were taken at a speed of 960 FPS using a digital camera (Model RX10‐IV, Sony of Canada Ltd., North York, ON, Canada). From these videos, the number of frames corresponding to ten wingbeats was quantified with the VSDC Free Video Editor v. 6.4.2 software (Flash‐Integro LLC). The wingbeat frequency (Hz) was then calculated as 10 × 960 FPS/number of frames for the ten beats. A minimum of 47 females per temperature treatment was filmed. All videos were made before 14:30 to allow the females to calm down and re‐acclimate before the start of the next test.

The influence of temperature on other components of flight performance was also quantified over a 19‐h period, starting at 15:00 and ending at 10:00 the next day. This quantification was automated, as each flight mill was equipped with a magnet on its arm, which triggered at each rotation a Hall effect sensor fixed to the mill axle. The Hall sensor was connected to a WP5‐6 rechargeable battery and a 4‐channel pulse data logger (Model UX120‐017; Hoskin Scientific Ltd., Saint‐Laurent, QC, Canada), which was set to record the number of rotations per second. At the end of flight tests, females were re‐weighed (final mass) to determine mass loss between the start and the end of the test. An in‐house Excel VBA program calculated flight propensity (proportion of females taking flight), flight onset time (min) after the start of the test, flight duration (h), distance flown (km), and flight speed (m/s; flight distance/flight duration). The growth chamber used in this experiment was not designed to control atmospheric pressure. Barometric pressure (kPa) in the chamber was thus determined by external atmospheric pressure, which we obtained from the MeteoMedia website (https://www.meteomedia.com/ca/meteo/quebec/quebec) every day at 10:30. This time of the day was chosen to account for typical daily variations in barometric pressure, which shows peaks at 10:00 and 22:00 and lower values at 4:00 and 16:00 (Barry and Chorley 2010). Females that laid eggs, detached, were damaged or died during the test were eliminated. Despite these losses, the flight performance of at least 54 females per temperature treatment was measured. The females were then frozen and stored at −17.3°C ± 0.1°C until dissected for morphological and physiological measurements.

#### 2.1.3 Morphological and Physiological Measurements

Females were dried for 24 h at 60°C (Shel Lab Gravity Convection Furnace Model 1330GM, Sheldon Manufacturing Inc., Cornelius, OR, USA), and weighed to obtain adult dry mass (mg). Their fore‐ and hindwings were carefully removed at the base and spread between two microscope slides. The ventral surface of each wing was then photographed at a magnification of 12× with a digital camera (model Infinity2; Luminera Corporation, Ottawa, ON, Canada), which was linked up to a microscope (model SteREO DiscoveryV20; Carl Zeiss Canada, North York, ON, Canada). The length (mm) and surface area (mm^2^) of each wing membrane were measured three times using ImageJ v1.49k (Schneider et al. 2012). As these repeated measurements of wing traits showed a high reliability, which ranged from 0.99 to 1.00, the average of the three measurements was used to estimate the length and area of the wings. In this study, the total surface area of all four wings, hereafter referred to as wing area, was used because a frenulum mechanically connects the fore‐ and hindwings, which beat synchronously and function as one large wing during flight in this insect. The wing aspect ratio was therefore calculated as the square of the wingspan (forewing length × 2) divided by the wing area, and the wing load as the female initial mass divided by the wing area (mg/mm^2^). The initial mass was used to minimize the error of the estimate, as we expected a non‐negligible loss of mass during the test that would vary with temperature, flight duration, or distance flown.

After removing the legs and head of the dry wingless moth, the abdomen and thorax were separated and weighed individually. Because most of the thorax is made up of the flight muscles (Kozlov 2012), the flight muscle ratio was calculated as thorax dry mass/female initial mass. All body parts were then weighed, placed in a 2 mL microcentrifuge tube (Neptune Microcentrifuge Tubes, VWR International, Mississauga, ON, Canada), and ground to extract the remaining lipids. Two consecutive 24‐h extractions were carried out by soaking the ground insect each time in 1.5 mL of a solution of ethyl acetate and methanol (2:1, v/v), a less toxic mixture than that used by Folch et al. (1957). After the second extraction, the pellet was dried at 60°C for 24 h and weighed. The remaining lipid mass was obtained by subtracting the dry mass of the pellet from the dry mass of the wingless moth.

### 2.2 Statistical Analysis

#### 2.2.1 Flight Performance

To limit the number of potential predictors describing wing and body traits, a prior selection was made based on cross‐correlations. Six morphological variables were preselected: female initial mass, forewing length, wing area, wing load, flight muscle ratio, and wing aspect ratio. Descriptive statistics of these six predictor variables and their cross‐correlations are provided in Tables A1 and A2 in the Appendix 1. Temperature and barometric pressure were always included as potential predictors in analyses. As the effect of temperature (*T*) was often curvilinear, a NLMIXED procedure was first performed to describe the relationship with a simple model (Table 1), which was then used as a temperature effect term instead of *T* in the model.

**TABLE 1:** Simplest models for temperature transformations, including the initial value of optimum *T*_0_, and Box‐Cox transformations to normalize flight component and cost variables.

Flight component	Temperature transformation	*T*_0_ (°C)	Transformation	Anderson‐Darling test	*p*
Onset time	1/*T* ^*2*^	—	$\psi^{\prime }=\left(\psi^{0.09}-1\right)/0.09$	0.60	0.117
Wingbeat	*T/*(*T*_*0*_ *+ T*)	24.6	—	1.52	< 0.05
Speed	(*T*−*T*_*0*_)^2^	25.8	Logarithmic	1.16	0.005
Duration	(*T*−*T*_*0*_)^2^	22.9	$\delta^{\prime }=\left(\delta^{0.34}-1\right)/0.34$	0.76	0.047
Distance	(*T*−*T*_*0*_)^2^	23.4	$\varphi^{'}=\left(\varphi^{0.2}-1\right)/0.2$	0.79	0.039
Mass loss	—	—	(*W*_*loss*_^0.54^–1)/0.54	0.26	0.724
Water loss	—	—	(W_water_^0.51^–1)/0.51	0.25	0.744
Fuel use	—	—	[(*W*_*fuel*_ + 0.179) ^0.55^–1]/0.55	0.22	0.837
Remaining lipids	—	—	Logarithmic	0.63	0.098

To select the best potential predictors among the six morphological and two abiotic variables for each flight performance component considered, Least Absolute Shrinkage and Selection Operator (LASSO) regression analyses (Tibshirani 1996) were performed with Proc HPGENSELECT for flight propensity (a binomial variable) and Proc GLMSELECT for all other flight components (SAS/9.4, SAS Institute Inc., Cary, NC, USA). The dependent variables (components of flight performance) were normalized as much as possible before LASSO analysis, using either logarithmic or optimal Box‐Cox transformations (Box and Cox 1964; Peterson 2021) (Table 1). No transformation was successful in normalizing wingbeat frequency, and the LASSO analysis was performed on the untransformed variable. In the case of flight speed, the logarithmic transformation was used although the resulting variable was not normally distributed. In either case, the departures from normality were not extreme, and the results of this preliminary analysis are believed to be adequately supported. Once the most likely predictors were selected, final parameter estimates were obtained with an NLMIXED procedure, using the untransformed data and their best‐fit distribution. Three unimodal probability distributions were tested because of their distinctive statistical properties. The normal, which is commonly used, is symmetrical, is valid over the range [−∞, ∞] and is particularly useful in the context of additive deviations (ε with mean 0 and variance *σ*_ε_^2^). The lognormal and Weibull are defined over the range [0, ∞], can be skewed (the Weibull can be positively or negatively skewed, and can even take exponential form), and are particularly well suited for multiplicative deviations when data exhibit heteroscedasticity: lognormal δ with mean 1 and variance *σ*_δ_^2^ or Weibull ξ with form *κ* and scale *λ* = 1/*Γ*(1 + 1/*κ*) ensuring a mean of 1. The distribution chosen for a given relationship was the one that showed the highest correlation between the observed and expected cumulative frequencies of individual deviations. Each variable was added to the model in the same order as it was selected in the LASSO regression, starting with a “null” model containing only the intercept and distribution parameter (when used) as predictors. The “support” for each model was expressed as a likelihood ratio that was calculated from improvements to the Akaike Corrected Information Criterion (AICc) relative to the best model (*e* ^−½ ΔAICc^) (Burnham et al. 2011). This support value ranges from very unlikely (0) to most likely (1) and was used to choose the form of the final models. In some cases, a model reduction (removal of the least likely predictor) yielded the best‐supported model.

#### 2.2.2 Flight Costs

To predict mass loss, water and fuel use during the flight test, as well as the remaining lipid mass, the following explanatory variables were preselected: temperature, female initial mass, forewing length, wing area, wing aspect ratio, wingbeat frequency, and distance flown.

**FIGURE A1:**
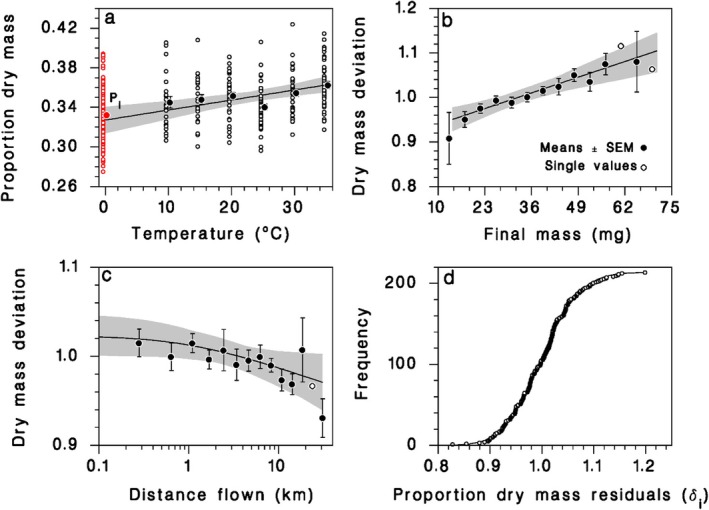
Relationship between proportion dry mass and (a) temperature (red symbols: estimated initial proportion dry weight), (b) final mass, (c) distance flow, and (d) cumulative frequency of individual deviations compared to a Weibull distribution. Solid lines: model curve. Shaded bands: 95% confidence on regression lines. Closed symbols: observed means ± SEM; open symbols: unique observations. Closed symbols: observed means ± SEM; open symbols: single values.

Loss of body mass was evaluated by the difference between the initial and final fresh masses, *W*_*b*_ and *W*_*a*_. The loss of body mass can result from both desiccation and fuel consumption. To distinguish the two, we first estimated the amount of water lost during the flight tests. To obtain this, the initial water mass of each female was estimated using three available mass measurements: the initial and final fresh masses, and the final dry mass *W*_*d*_. The final proportion of dry mass was calculated as *P*_*d*_ = *W*_*d*_/*W*_*a*_. Assuming that females flying the least (or not at all) at 10°C lost the least water, we related *P*_*d*_ to temperature *T*, flight distance *D'*, and final body mass *W*_*a*_ using the model *P*_*d*_ = (*p*_1_ + *p*_2_ *T* + *p*_3_ *D'* + *p*_4_ *W*_*a*_) *δ* (see Figure A1a in Appendix 1). Using this relationship and its residuals *δ*_*i*_, the initial dry mass proportion *P*_*i*_ of each individual was calculated by setting *T* = 0°C and *D =* 0 km, yielding *P*_*i*_ = *δ*_*i*_ (0.3 + 0.009 *W*_*a*_) (red symbols at 0°C in Figure A1a). With these estimates of *P*_*i*_, we calculated the amount of water lost during flight as *W*_*water*_ = *W*_*a*_ (*P*_*d*_−*P*_*i*_). This procedure yielded realistic estimates of water loss, with only one individual value < 0 (−0.0159 mg), which was set to 0.05 mg for the determination of the final model of water loss. The fuel mass consumed during flight was estimated with *W*_*fuel*_ = *W*_*b*_−*W*_*a*_−*W*_*water*_. Three of the 214 values of fuel use thus calculated were < 0 (minimum −0.18 mg), all occurring at 10°C. For LASSO analysis, all fuel mass values were incremented by 0.18, but no transformation was needed for final analysis with NLMIXED. Here, power law equations were used. Selection of probable predictors and building of final models was carried out as described in the previous section.

In the figures, the relationships between flight propensity and predictors represent the effect of each explanatory variable calculated at the average value of other model predictors. For all other flight and cost components, the regression functions against temperature are based on full prediction equations using the mean values of all other predictors. The effect of predictors *X* other than temperature was represented as relative “deviations”. Observations were calculated as $Y_{i}^{\prime }=Y_{i}/f\left(X_{k},{\bar{X}}_{j}\right)$ where *i* refers to an individual observation, and $f\left({\bar{X}}_{k},X_{j}\right)$ is the regression model relating *Y* to all predictors *X*, using individual values of *j* terms and the mean of the *k* term (the effect of which is being represented). An average *Y′* was then calculated for 14 or 15 classes over the range of predictor *k*. Predicted lines are ${\hat{Y}}_{k}=f\left({\bar{X}}_{j},X_{k}\right)/f\left(\bar{X}\right)$.

For all final models, 95% regression confidence bands were calculated using the Monte Carlo method (Mazumdar 1995). Parameter values are varied at random (according to their standard error), and the likelihood ratio between the likelihood of the best model and the likelihood of the model using randomized parameter values is calculated. If the likelihood ratio test (a chi‐square statistic) is nonsignificant, the predicted values are stored. It is the envelope between lower and highest predicted values that forms the confidence band. The number of random replicates was varied (between 1000 and 10,000) so that ~100 equally likely sets of predictions were obtained.

## 3 Results

### 3.1 Flight Performance

**FIGURE 1:**
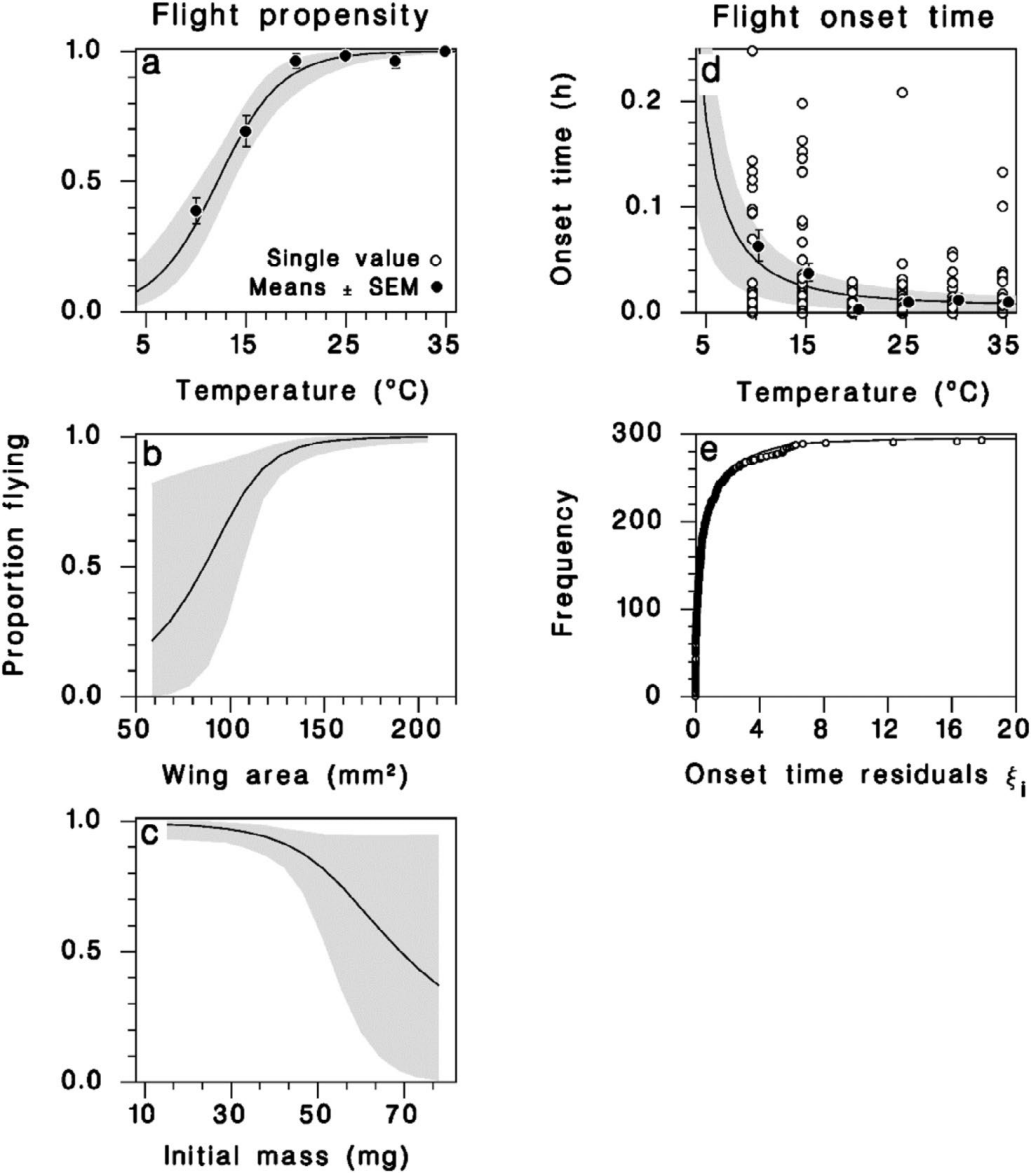
Flight initiation relationships. Equation (1) (Table 2) predicting flight propensity from (a) temperature, (b) wing area, and (c) initial mass, each represented at the average value of the other predictors. Equation (2) (Table 2) predicting flight onset time from (d) temperature, and (e) the frequency distribution of residuals from the final regression described by a Weibull distribution. Solid lines: Mean model prediction. Shaded bands: 95% confidence on regression lines. Closed symbols: Observed means ± binomial SEM in (a) and ± SEM in (d); open symbols: Single values.

Temperature had a strong curvilinear influence on flight propensity, as the proportion of females that flew increased from 37.5% at 10°C to over 90% at 20°C and above (Figure 1a; Equation (1) in Table 2). An increase in wing area increased flight propensity, while initial mass had the opposite effect (Figure 1b,c). The final model with three predictors was far more likely than a model using only temperature as a predictor (likelihood ratio = *e* ^½ΔAICc^ = 42,192; Table 2). At low temperatures (15°C and 10°C), females also took 2.5 to 5 times longer to start flying than those tested at warmer temperatures (Figure 1d; Equation (2) in Table 2). Residuals from this relationship clearly followed a Weibull distribution that was nearly exponential (parameter *κ* ≤ 1; Figure 1e).

**TABLE 2:** Equations, incremental fits, and final models resulting from Proc NLMIXED for flight behavior components. Support: How much less likely the current model is, compared to the final model with the lowest AICc (Burnham et al. 2011).

Model^a^		Incremental model building			Final model		
		Term^a^	AICc	Support	Parameter^a^	Estimate	SE
*Propensity* $P_{f}=\frac{1}{1+e^{-\left(p_{1}+p_{2}T+p_{3}S_{\mathrm{tot}}+p_{4}W_{b}\right)}}$ *N* = 379, *R* ^2^_adj_ = 0.41	Equation (1)	Intercept	403.0	0.00	*p*_1_	−8.523	1.406
		*T*	262.1	0.00	*p*_2_ (*T*)	0.307	0.039
		*A*_*p*_	263.6	0.00	*p*_*3*_ (*S*_*tot*_)	0.064	0.017
		*S*_*tot*_	251.7	0.00	*p*_4_ (*W*_*b*_)	−0.090	0.032
		*W*_*b*_	242.7	0.40			
		*A*_*p*_ removed	240.8	1.00			
*Onset time* $\psi_{i}^{\prime }=\left[p_{1}+p_{2}/T^{2}\right]\xi_{i}$ *N* = 295, *R* ^2^ = 0.14	Equation (2)	Intercept, *κ*	2495.1	0.00	*p*_1_	0.005	0.002
		1/*T* ^*2*^	2453.6	1.00	*p*_2_ (1/*T* ^2^)	4.465	0.978
		*τ*_*t*_	2455.6	0.36	*κ*	0.578	0.025
		*ω*_*tot*_	2457.4	0.15			
*Wingbeat frequency* $\nu_{i}=\left[p_{1}+p_{2}\left(\frac{T}{T_{0}+T}\right)+p_{3}L_{a}\right]\xi_{i}$ *N* = 215, *R* ^2^_adj_ = 0.69	Equation (3)	Intercept, *κ*	1233.3	0.00	*p*_1_	4.178	5.065
		*T*_0_, *T*	943.3	0.52	*T*_0_	23.242	7.337
		*L*_*a*_	942.0	1.00	*p*_2_ (*T*)	77.331	3.813
		*ω*_*tot*_	942.0	0.97	*p*_3_ (*L*_*a*_)	−0.446	0.241
					*κ*	10.908	0.629
*Speed* $\log_{10}\left(\eta_{i}\right)=p_{1}+p_{2}{\left(T-T_{0}\right)}^{2}+p_{3}L_{a}+\varepsilon_{i}$ *N* = 282, *R* ^2^_adj_ = 0.21	Equation (4)	Intercept, *σ* ^2^_ε_	−74.1	0.00	*p*_1_	−1.213	0.124
		*T*_0_, *T*	−103.3	0.00	*T*_0_	27.117	1.023
		*L*_*a*_	−135.9	0.96	*p*_2_ (*T*)	−0.001	0.0001
		*τ*_*T*_	−136.0	1.00	*p*_3_ (*L*_*a*_)	0.077	0.013
					*σ* ^2^	0.035	0.003
*Duration* $\delta_{i}=\left[p_{1}+p_{2}{\left(T-T_{0}\right)}^{2}+p_{3}\tau_{t}+p_{4}S_{\mathrm{tot}}+p_{5}A_{p}+p_{6}\omega_{\mathrm{tot}}\right]\xi_{i}$ *N* = 295, *R* ^2^_adj_ = 0.18	Equation (5)	Intercept	5528.4	0.00	p_1_	−24.197	1.003
		*T*_0_, *T*	5469.9	0.00	*T*_0_	23.718	0.271
		*τ*_*T*_	5467.3	0.00	*p*_2_ (*T*)	−0.012	0.001
		*S*_*tot*_	5463.9	0.00	*p*_3_ (*τ*_*T*_)	−29.721	9.141
		*A*_*p*_	5456.4	0.00	*p*_4_ (*S*_*tot*_)	0.016	0.004
		*ω*_*tot*_	5442.5	1.00	*p*_5_ (*A*_*p*_)	0.281	0.008
					*p*_6_ (*ω*_*tot*_)	−9.754	1.650
*Distance* $\varphi_{i}=\left[p_{1}+p_{2}{\left(T-T_{0}\right)}^{2}+p_{3}L_{a}+p_{4}A_{p}\right]\xi_{i}$ *N* = 295, *R* ^2^_adj_ = 0.17	Equation (6)	*i*, *κ*	4834.6	0.00	*p*_*1*_	−9.912	0.796
		*T*_0_, *T*	4774.0	0.00	*T*_*0*_	23.551	0.255
		*τ*_*T*_	4772.9	0.01	*p*_*2*_ (*T*)	−0.024	0.003
		*L*_*a*_	4766.5	0.18	*p*_*3*_ (*L*_*a*_)	0.382	0.082
		*A*_*p*_	4764.0	0.64	*p*_*4*_ (*A*_*p*_)	0.109	0.007
		*τ*_*T*_ removed	4763.1	1.00	*κ*	0.691	0.034

Abbreviations: *N*, number of females; *R* ^2^_adj_, Adjusted for number of parameters and sample size.

^a^ *p*_1_‐*p*_6_, *T*_0_, *κ* and *σ* ^2^_ε_ are parameters; *i* refers to individual females; *T, A*_*p*_, *W*_*b*_, *τ*_*t*_, *S*_*tot*_, *ω*_*tot*_, and *L*_*a*_ are the temperature, barometric pressure, initial mass, flight muscle ratio, wing area, wing loading and forewing length, respectively; *ξ* is a Weibull random variable (the exponential distribution is a special case with *λ* = *κ* = 1), and *ε*_*i*_ is a normal random variable with mean *E*(*ε*) = 0 and variance *σ* ^2^_ε_.

**FIGURE 2:**
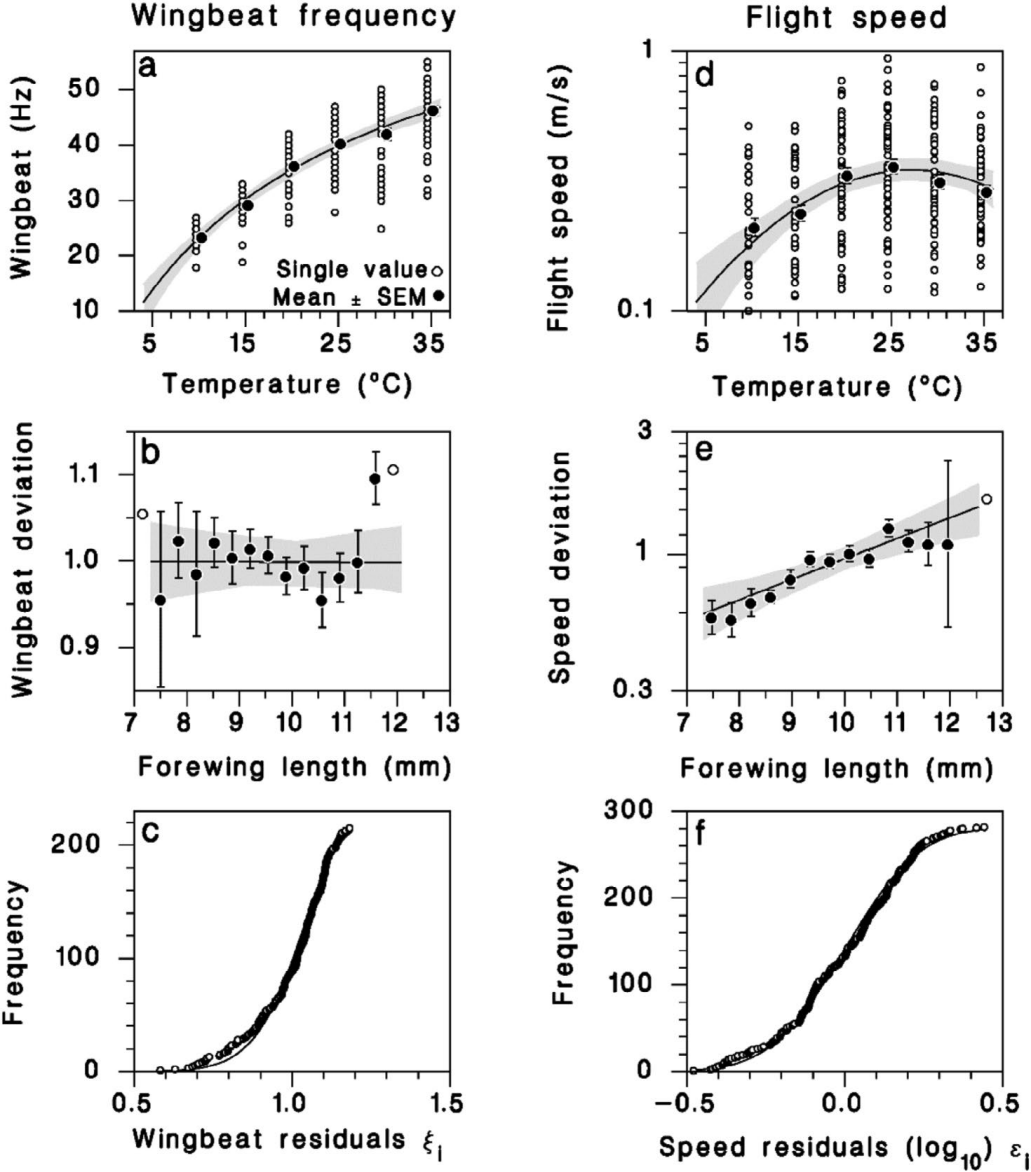
Propulsion relationships. Equation (3) (Table 2) predicting wingbeat frequency from (a) temperature, (b) forewing length (deviations after accounting for *T* effect), and (c) cumulative frequency of individual residuals compared to a Weibull distribution. Equation (4) (Table 2) predicting flight speed from (d) temperature, (e) forewing length (deviations after accounting for *T* effect), and (f) cumulative frequency of individual residuals (logs) compared to a normal distribution. Solid lines: Mean model predictions. Shaded bands: 95% confidence on regression lines. Closed symbols: Observed means ± SEM; open symbols: Single values.

Wingbeat frequency increased from 23.4 ± 0.3 Hz at 10°C to 46.0 ± 0.8 Hz at 35°C, in diminishing return fashion (Figure 2a; Equation (3) in Table 2). The wingbeat frequency also decreased slightly, but significantly, over the range of observed forewing lengths, by a barely detectable 2 Hz. Yet, the inclusion of this term doubled the likelihood of the model (Figure 2b, Table 2). The residuals were best described by a Weibull distribution (Figure 2c). Flight speed varied parabolically with temperature, reaching a maximum at *T*_0_ = 27.1°C ± 1.0°C (Figure 2d; Equation (4) in Table 2). In addition to the effect of temperature, flight speed increased with forewing length (Figure 2e). This flight component showed strong variation between individuals, in the range of one order of magnitude, and the cumulative frequency of individual deviations followed a lognormal distribution (Figure 2f).

**FIGURE 3:**
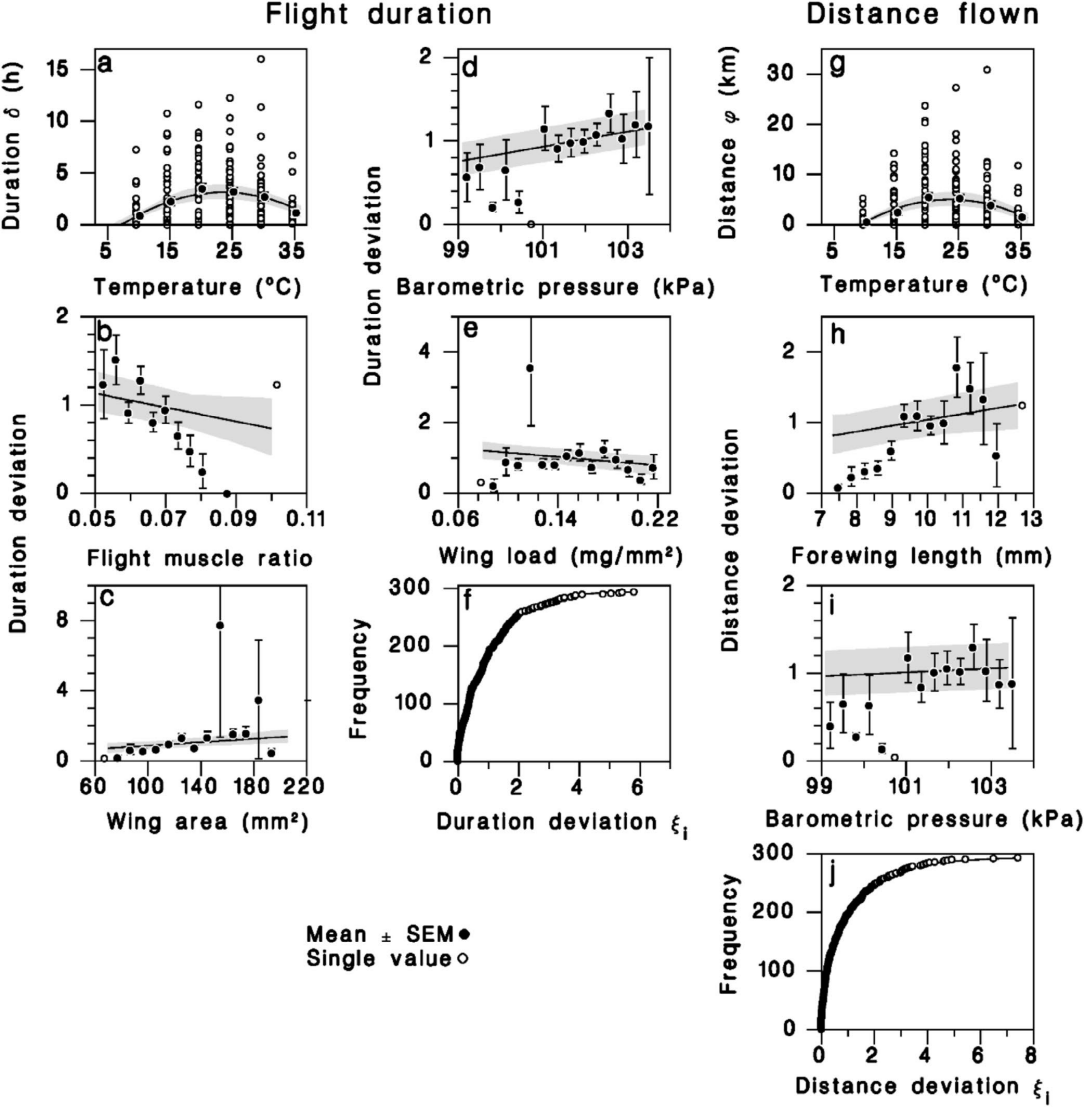
Flight endurance relationships. Equation (5) (Table 2) predicting flight duration from (a) temperature, (b) flight muscle ratio, (c) wing area, (d) barometric pressure, (e) wing load, and (f) cumulative frequency of individual deviations compared to an exponential distribution. Equation (6) (Table 2) predicting flight distance from (g) temperature, (h) forewing length, (i) barometric pressure, and (j) cumulative frequency of individual deviations compared to a Weibull distribution. Solid lines: Model curve. Shaded bands: 95% confidence on regression lines. Closed symbols: Observed means ± SEM; open symbols: Single values.

Flight duration (Figure 3a) and distance flown (Figure 3g) also showed parabolic relationships with temperature, peaking between 23°C and 24°C (*T*_0_ of Equations (5) and (6) in Table 2) and dropping rapidly at both ends of the response curve to zero at temperatures of about 7°C and 39°C. Although the mean flight duration was near 3.5 h at 20°C and 25°C (Figure 3a), some females tested at temperatures between 15°C and 30°C flew for > 10 h. In addition to the temperature effect, flight duration decreased with flight muscle ratio and wing load (Figure 3b,e), while it increased with wing area and barometric pressure (Figure 3c,d; Equation (5) in Table 2). However, these predictors did not much improve the predictions based solely on temperature, which remains the variable with the greatest predictive power. Flight duration varied greatly from female to female, and these individual variations were well described by an exponential distribution (Figure 3f). Females flew an average distance of just over 5 km at 20°C and 25°C (Figure 3g). A record distance of 31 km was observed at 30°C. With temperature, two other predictors had a positive effect on the distance flown: forewing length and barometric pressure (Figure 3h,i; Equation (6) in Table 2). An exponential‐like Weibull distribution described the individual variation in distance flown relative to the mean (Figure 3j). It is important to note that 16.4% of females died during the test at 35°C, compared to only 1.1% in other temperature treatments. In addition, forewing length and wing surface were correlated with each other (*F*_1,394_ = 4839.75, *p* < 0.0001, *r* ^2^ = 0.93) and with initial female weight (forewing: *F*_1,386_ = 1326.57, *p* < 0.0001, *r* ^2^ = 0.78; wing area: *F*_1,384_ = 2098.76, *p* < 0.0001, *r* ^2^ = 0.85), in agreement with previously published work (Van Hezewijk et al. 2018). In some of our models, we used total wing area (*S*_*tot*_) as a predictor. Other models in the literature use the area of a single forewing (*S*_*f*_). In our data, the relationship between them is linear: *S*_*tot*_ = 3.763 *S*_*f*_ + 2.647 (*F*_1,394_ = 28342.10, *p* < 0.0001, *r* ^2^ = 0.987).

### 3.2 Flight Costs

**FIGURE 4:**
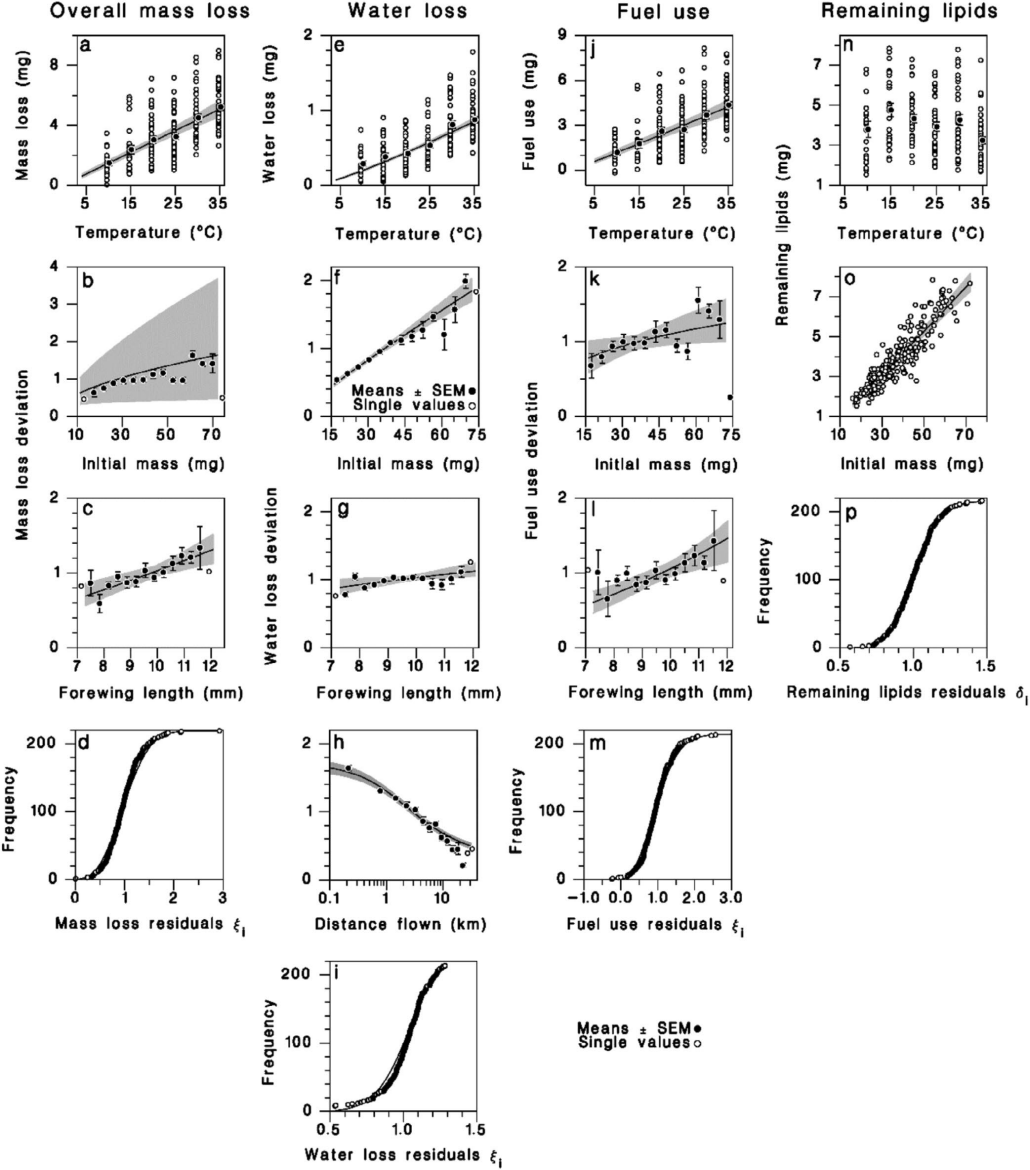
Flight cost relationships. Equation (7) (Table 3) predicting body mass loss from (a) temperature, (b) initial mass, (c) forewing length, and (d) distribution of individual variation compared to a Weibull distribution. Equation (8) (Table 3) predicting water loss from (e) temperature, (f) initial mass, (g) forewing length, (h) distance flown, and (i) cumulative frequency of individual variation compared to a Weibull distribution. Equation (9) (Table 3) predicting fuel use from (j) temperature, (k) initial mass, (l) forewing length, and (m) cumulative frequency of individual deviations compared to a Weibull distribution. Equation (10) (Table 3) predicting remaining lipid mass from (n) temperature, (o) initial mass, and (p) cumulative frequency of individual deviations compared to a lognormal distribution. Solid lines: Model curve; Shaded bands: 95% confidence on regression lines. Closed symbols: Observed means ± SEM; open symbols: Single values.

As expected, females lost body mass during the flight test. Mass loss increased with temperature, from an average of 1.4 ± 0.1 mg at 10°C (or 3.6% ± 0.3%) to 4.5 ± 0.1 mg (or 5.7% ± 0.6%) at 35°C (Figure 4a; Equation (7) in Table 3). The mass loss of large females was proportionally less than that of small ones, and the exponent of the relationship (0.44 ± 0.17) did not differ significantly from either 0.75 or 0.66, two exponents predicted by the metabolic theory of ecology (Glazier 2010; Niven and Scharlemann 2005) (hypoallometry: *t* < −1.86, df = 219, *p* > 0.05) (Figure 4b; Table 3). Body mass loss also increased with forewing length (Figure 4c; Equation (7) in Table 3). Individual deviations from mean mass loss were well described by a Weibull distribution (Figure 4d).

**TABLE 3:** Equations, incremental fits, and final models resulting from the Proc NLMIXED for flight physiological costs. Support: How much less likely the current model is, compared to the best model (with the lowest AICc) (Burnham et al. 2011).

Model^a^		Incremental model building			Final model		
		Term^a^	AICc	Support	Parameter^a^	Estimate	SE
*Body mass loss* (*W*_*loss*_) $W_{\text{loss}}=p_{1}\;T^{p_{2}}\;{W_{b}}^{p_{3}}\;{L_{a}}^{p_{4}}\xi$ *N* = 219, *R* ^2^_adj_ = 0.6	Equation (7)	*p*_1_, *κ*	2599.1	0.00	*p*_1_	0.002	0.002
		*T*	2516.6	0.00	*p*_2_ (*T*)	0.992	0.069
		*W*_*b*_	2440.0	0.33	*p*_3_ (*W*_*b*_)	0.442	0.165
		*L*_*a*_	2437.7	1.00	*p*_4_ (*L*_*a*_)	1.260	0.575
		*D*	2439.3	0.46	*κ*	2.703	0.134
*Water mass loss* (*W*_*waterLoss*_) $W_{\text{waterLoss}}=p_{1}\;T^{p_{2}}\;{D^{\prime }}^{p_{3}}\;{W_{b}}^{p_{4}}\;{L_{a}}^{p_{5}}\xi$ *N* = 214, *R* ^2^ = 0.93	Equation (8)	*i*, *κ*	1775.1	0.00	*p*_*1*_ (*i*)	0.0003	0.0001
		*T*	1687.1	0.00	*p*_2_ (*T*)	1.178	0.030
		*D*	1610.6	0.00	*p*_3_ (*D*)	−0.963	0.038
		*W*_*b*_	1259.9	0.20	*p*_4_ (*W*_*b*_)	0.916	0.054
		*L*_*a*_	1256.7	1.00	*p*_*5*_ (*L*_*a*_)	0.485	0.204
		*ν*	1257.4	0.69	*κ*	7.223	0.418
*Fuel use* (*W*_*fuel*_) $W_{\text{fuel}}=p_{1}\;T^{p_{2}}\;{W_{b}}^{p_{3}}\;{L_{a}}^{p_{4}}\xi$ *N* = 214, *R* ^2^_adj_ = 0.5	Equation (9)	*i, κ*	2499.3	0.00	*p*_*1*_ (*i*)	0.0007	0.0008
		*T*	2431.5	0.00	*p*_2_ (*T*)	1.037	0.085
		*W*_*b*_	2381.7	0.00	*p*_3_ (*W*_*b*_)	0.328	0.206
		*D*	2380.8	0.01	*p*_4_ (*L*_*a*_)	1.687	0.718
		*L*_*a*_	2379.1	0.77	κ	2.230	0.115
		*D* removed	2378.6	1.00			
*Remaining lipid mass* (*W*_*lipids*_) $W_{\text{lipids}}=p_{1}\;{\omega_{b}}^{p_{2}}\delta$ *N* = 216, *R* ^2^_adj_ = 0.85	Equation (10)	*i*, *σ*_ε_^2^	201.9	0.00	*p*_*1*_ (*i*)	0.086	0.009
		*W*_*b*_	−207.2	1.00	*p*_2_ (*W*_*b*_)	1.051	0.030
					*σ*_ε_^2^	0.022	0.0001

Abbreviations: *N*, Number of females; *R* ^2^_adj_, Adjusted for number of parameters and sample size.

^a^ *p*_1_‐*p*_4_, *κ* and *σ* ^2^_ε_ are parameters; *T, W*_*b*_, *L*_*a*_, *S*_*tot*_, *α*_*tot*_, *ν, and D'* are respectively the temperature, initial mass, forewing length, wing area, aspect ratio, wingbeat frequency, and distance flown *D'* = *ln*(*D* + e) so that *D'* = 1 when flight distance *D* = 0 in all models except *P*_*d*_ where *D'* = *ln*(*D* + 1); *ξ* is a Weibull random variable (the exponential distribution is a special case with *λ* = *κ* = 1), and *δ* is a multiplicative individual variation term with a lognormal distribution with mean 1 and variance *σ*_δ_^2^.

The amount of water lost by females during the flight tests increased with temperature and initial mass (Figure 4e,f; Equation (8) in Table 3), with females losing water in direct proportion to their initial mass (isometry: *t* = 0.50, df = 211, *p* > 0.50). During the flight test, females lost on average 0.59 ± 0.02 mg water, or about 2.6% of their initial water content (22.67 ± 0.53 mg). Water loss also increased with forewing length, while it decreased with distance flown (Figure 4g,h). Residuals followed a Weibull distribution (Figure 4i). Because water loss was small, most of the body mass loss measured during flight tests can be attributed to fuel use (use of lipids and carbohydrates = mass loss−water loss).

Fuel use increased with temperature, initial mass, and forewing length (Figure 4j–l; Equation (9) in Table 3). Smaller females consumed more fuel in proportion to their mass than their larger counterparts, as indicated by the exponent of the relationship [*p*_3_ = 0.328 ± 0.206 in Equation (9)] significantly smaller than 0.75 (hypoallometry: *t* = −2.05, *N* = 214, *p* = 0.04) but not 0.66 (*t* = −1.65, *N* = 214, *p* = 0.1). The cumulative frequency of individual deviations from mean fuel use was well described by a Weibull distribution (Figure 4m). Fuel use averaged 2.94 ± 0.12 mg, which represented 40% of the fuel mass available before the test (fuel mass before the test = *W*_*b*_−*W*_*a*_−*W*_*waterLoss*_).

The mass of lipids remaining after the tests averaged about 4 mg and was not dependent on temperature (Figure 4n; Equation (10) in Table 3). It was directly proportional to initial mass and represented 8.6% of total female initial mass (isometry: *t* = 1.69, *N* = 216, *p* = 0.093) (Figure 4o). Individual variance in remaining lipid mass was small (*σ*_ε_^2^ = 0.018 ± 0.002) and followed a lognormal distribution (Figure 4p).

## 4 Discussion

Thermal performance response functions are crucial for the development of flight models that estimate the movement of individual insects under specific environmental conditions and can inform studies of insect populations over time and, especially, in response to climate change (Hillaert et al. 2015; Sinclair et al. 2016; Travis et al. 2013). In addition to developing thermal performance functions, this study aimed to determine the underlying mechanisms of spruce budworm flight. It is likely the first to integrate temperature and functional morphology into models of flight performance and costs in an insect.

Surprisingly, females of the spruce budworm were active in a much wider temperature range than previously known (14.4°C to 29.5°C) (Greenbank et al. 1980). Most females flew at the highest temperatures (30°C and 35°C), suggesting that a warmer climate is not likely to reduce the flight propensity of this species. Although flight propensity decreased below 20°C, it was not completely inhibited. Tethered moths do not have to lift themselves to start flying, but they must still produce the vertical force necessary to subtract their mass from gravity (Naranjo 2019). The reduced propensity to fly in cold weather may thus be due to the inability of flight muscles to reach the minimum temperature and thus the wingbeat frequency necessary to lift the mass of the insect (Heinrich 1974, 1989). However, 37.5% of budworm females flew at 10°C. As the wings of many females shivered when they were placed on ice packs, some larger moths were likely able to warm up their flight muscles using the wing shivering mechanism that is well known in moths (Heinrich 1974, 1989). This mechanism is also consistent with the observed delays in the flight onset time at 10°C and 15°C, as the period of muscle warming to bridge the gap between the minimum temperature for flight and the air temperature is longer in cold weather (Dorsett 1962; Heinrich and Bartholomew 1971). These findings suggest that spruce budworm females could be capable of endo‐thermogenesis, but this ability will need to be confirmed by experiments aimed at quantifying thoracic temperature at takeoff and during flight.

Flight propensity of budworm females increased with wing area and decreased with female mass, which are the two components of wing load. The response functions to these two traits define a wide range of initial morphologies that are conducive to flight activity. However, flight propensity decreased as the mass of females increased relative to their wing area, suggesting that there is a maximum threshold of wing load (mass/area) beyond which the probability of flying drops. As mating of budworm females occurs shortly after emergence (Outram 1971) and stimulates egg maturation (Wheeler 1996), it is possible that the egg load may unbalance the morphology of gravid females to the point where it exceeds this presumed threshold wing load. It would thus be to restore the initial biomechanical balance conferred by their morphology that budworm females lay a part of their egg complement before dispersing in nature (Greenbank et al. 1980; Rhainds and Kettela 2013; Sanders and Lucuik 1975). The mean mass of virgin females tested in this study was comparable to that of wild gravid females that flew after laying a part of their eggs: 35.1 ± 0.6 mg after flight in this study compared with 35 mg reported by Greenbank et al. (1980); or 12.7 ± 0.3 mg compared with 11 mg dry mass after flight reported by Rhainds and Kettela (2013). If the activity of females depends mainly on their morphology, we are confident that our flight results with virgin females can be applied to wild mated females.

As expected, the flight components related to speed and endurance in budworm females decreased when the thermal conditions deviated from an optimum (Kingsolver 2009; Sinclair et al. 2016). The optimum of most flight components was estimated at about 23°C, except for speed, which peaked at 27°C (see *T*_0_ in Table 2). Under optimal conditions, the average distance flown was 5 km, but some females traveled more than 20 km in still air. This daily flight capacity is sufficient to track the northward movement observed over the past decades of the climate envelope, where conditions are suitable for the survival and reproduction of the spruce budworm (Boulanger et al. 2025). The budworm females covered these distances at an average speed of 0.4 m/s, which is within the range of speeds estimated during the ascending phase of the migratory flight of noctuid moths and acridid grasshoppers (Reynolds et al. 2009). Therefore, these flight distances and speeds are more than sufficient to reach the air currents of the lower troposphere where the wind‐borne dispersal of the spruce budworm occurs (Boulanger et al. 2017). As wind speeds in this layer of the troposphere can reach 20 to 40 km/h and temperatures can vary from 16°C to 26°C as the temperature inversion progresses (Boulanger et al. 2017), budworm females could travel 70 to 140 km during the 3.5 h that their daily flight lasts on average, and the females having flown more than 10 h could cover at least 200 to 400 km. These estimates are comparable to those of wind‐borne flights observed in nature (Boulanger et al. 2017; Dobesberger et al. 1983; Greenbank et al. 1980; Larroque et al. 2019) and simulated by Garcia et al. (2022, 2024), although flight distance, speed, and duration may be underestimated on flight mills (Naranjo 2019; Ribak et al. 2017).

The wingbeat frequency of budworm females increased with temperature from 23.4 to 46.0 Hz, which is consistent with previously published estimates for this species (Greenbank et al. 1980). As expected, wingbeat frequency tended to plateau at higher temperatures, because there is a maximum limit to the rate of contraction of flight muscles (Dudley 2000). However, our results suggest that the atmospheric transport model of the spruce budworm (ATM) (Garcia et al. 2022, 2024; Régnière, Delisle, et al. 2019) underestimates the wingbeat frequency at low temperatures and overestimates it at high temperatures. As this flight component is at the heart of the ATM, the integration of these new data, including the relationship between temperature and flight propensity, would improve the predictions of wind‐borne flight of spruce budworm by this model.

At the coldest temperatures, the poor flight performance of budworm females can be attributed to the difficulty of maintaining the minimum temperature for flight over a long period (Church 1960b; Heath and Adams 1967), while the low frequency of wingbeat limits heat production by flight muscles (Heath and Adams 1967; Heinrich and Bartholomew 1971). The cost of maintaining this minimum temperature for flight should be high in cold weather (Srygley 1994), but the low proportion of females that flew at cold temperatures did not allow us to demonstrate this in the model of fuel use. However, the lipid mass remaining after the test was similar in females that flew at cooler and warmer temperatures, although the former flew shorter distances and for a shorter time. As the main fuel used during flight is generally lipids (Arrese and Soulages 2010; Beenakkers et al. 1984), this finding suggests that the cost of flight was indeed high at low temperatures. Our results also indicate that budworm females flying at 15°C or less could reach the lower troposphere but would not benefit from wind‐borne flight for long. Indeed, convective cooling can dissipate the heat produced by muscles, despite the thick insulating layer of hair‐like scales that covers the thorax of the spruce budworm (Church 1960b; Heath and Adams 1967).

The insulating layer of scales that limits heat dissipation can become an issue at high temperatures. Heat build‐up may lead to overheating (Heath and Adams 1967; Mattila 2015), which could explain the mortality of budworm females observed at 35°C. If this is indeed the case, this high temperature would approach the upper limit of thermal tolerance for flight of this species (Kingsolver 2009; Sunday et al. 2014). The decline in flight activity at warm temperatures would then be a behavioral adjustment to prevent overheating, as has been suggested in other species (Hanegan and Heath 1970b; Sunday et al. 2014). In any case, the decline in the flight activity of spruce budworm cannot be attributed to dehydration (Church 1960a) or depletion of energy resources (Beenakkers et al. 1984; van Handel 1974), as only 2.6% of the initial water content and 40% of the initial fuel mass were lost during the tests. However, another explanation for the decline in flight activity emerges from our results. First, the increase in metabolism with temperature led to increasing mass losses in females, which was mainly due to fuel consumption. Second, the mass of remaining lipids was independent of temperature and proportional to the initial mass of the female. Together, these findings suggest that budworm females allocate a daily energy budget to flight, which is not fixed as it is in *Hyalophora cecropia* (Hanegan and Heath 1970a), but proportional to their initial mass. A decrease in flight duration and distance would then result from a faster depletion of the energy allocated to flight in the daily budget at high temperatures. Some of the remaining lipids are likely sequestered in the ovaries and mature eggs. Lipids are also needed to maintain the body and fly locally in search of oviposition sites. Moreover, the adult spruce budworm is not known to feed. Because these moths cannot replenish lipid reserves by feeding, our results on fuel use and remaining lipids suggest that females will rarely undertake a second long‐distance dispersal flight, as observed by Greenbank et al. (1980). Therefore, climate warming is expected to have a negative impact on the flight endurance of spruce budworm, which may decrease the contribution of southern populations to the spread of outbreaks and to the synchrony of its metapopulations (Larroque et al. 2019, 2020; Régnière et al. 2012, 2013; Régnière and Nealis 2019). This impact could be aggravated if warm temperatures during the insect's development induce changes in the morphological traits associated with flight (Sánchez‐García et al. 2024).

Forewing length was the most variable morphological trait and the only one appearing in the models of propulsion and distance flown in budworm females. Because the wing tip moves faster than the base, long wings can move air at greater relative speed and thus generate more lift power per unit area with each wingbeat (Dudley 2000; Ellington 1984, 1999). This improved efficiency explains the decrease in wingbeat frequency and the increase in flight speed and distance with forewing length in budworm females and other species of Lepidoptera (Berwaerts et al. 2002; Flockhart et al. 2017). Therefore, long‐winged females of the spruce budworm would be more likely to disperse over long distances by self‐propelled flight, as is the case with migratory populations of the monarch, *Danaus plexippus* (L.) (Dockx 2007; Flockhart et al. 2017). Although long wings can reduce drag and therefore the costs of flight (Dudley 2000), fuel use of budworm females increased with forewing length, probably because long‐winged females flew longer distances (Hammond and Fescemyer 1987; Roff 1991). Water loss also increased with forewing length, as energy consumption increases the respiratory rate (Mellanby 1935). However, water loss decreased with distance flown to hydric quasi‐homeostasis at distances exceeding 10 km. This surprising result suggests that the water was very well conserved, or its loss was compensated by water produced metabolically during long‐range flights, as occurs in *Hyalophora cecropia* (L.) (Hanegan and Heath 1970a). On one hand, water could have been better conserved by adjusting the rate of gas exchange to flight distance (Oladipupo et al. 2022), a process that we did not quantify. On the other hand, the contribution of distance to the variation of fuel consumption would have been sufficient to explain the small decrease in water loss with distance due to lipid oxidation, but distance was removed from the final model of fuel use. Therefore, our results do not completely exclude that the production of metabolic water played a role in the water budget of this insect during flight. The mechanism underlying this variation in water loss remains to be elucidated in the spruce budworm. Overall, our results indicate that long‐winged females of the spruce budworm have more efficient propulsion allowing them to travel a greater distance per unit of time, while saving the water needed for their eggs to mature (Chapman 2013).

Flight duration increased with wing area, as large wings promote lift and should also reduce flight costs (Altizer and Davis 2010; Angelo and Slansky 1984). As wing length and area are correlated with the mass of budworm females, long‐ and broad‐winged females would be heavier. We found that large budworm females consumed less fuel relative to their mass than their smaller counterparts. This finding supports the hypothesis that mass‐specific energy costs of flight decrease with size (Roff 1991). Flight duration also decreased with the ratio of flight muscle to body mass. A large flight muscle ratio allows fast accelerations and good maneuverability (Chai and Srygley 1990; Marden 2000), which can be useful for moving through the structural complexity of the forest. However, budworm females only need to produce a minimal amount of thrust force to maintain their lift during dispersal flights above the canopy (Greenbank et al. 1980; Marden 2000). It would then be more advantageous for budworm females to carry fuel and eggs than a muscle mass that exceeds the minimum required to maintain lift (Marden 1987). But the transport of fuel and eggs would be limited by the wing area of the female, because flight duration decreases with wing load. Therefore, gravid females with a large egg load are less able to lift off and will not be able to fly for long. This may explain the observation that gravid females of the spruce budworm must lay a part of their eggs to restore their biomechanical balance before undertaking long‐range dispersal (Greenbank et al. 1980; Rhainds and Kettela 2013; Sanders and Lucuik 1975).

The flight endurance of budworm females increased with barometric pressure. High barometric pressures are generally associated with clear weather and low to moderate winds (Weisser et al. 1997), which correspond to the weather conditions observed during wind‐borne flights of the spruce budworm (Boulanger et al. 2017; Morris 1963). In contrast, precipitation and strong winds that occur at low barometric pressure tend to stop the flight activity of the spruce budworm (Morris 1963). Such poor conditions can also lead to high mortality in small insects (Weisser et al. 1997).

Our results reveal that forewing length and wing surface are the key morphological traits to explain the biomechanics of the flight performance in spruce budworm. These morphological traits favor the propulsion and the lift capacity of females, allowing them to have greater flight endurance. Long wings can also give budworm females greater stability by reducing the rolling moment (Le Roy et al. 2019) when dispersing in the lower troposphere, where wind speeds can exceed 20 km/h (Boulanger et al. 2017). These morphological traits are also characteristic of larger females. Large budworm females benefit from more energy reserves that can be allocated daily to flight and a relative energy saving during flight due to their mass. For the spruce budworm, the initial energy reserve and its economy can be decisive, because wind‐borne flights are made in a single night without the possibility of refueling (Boulanger et al. 2017; Greenbank et al. 1980). Together, wing morphology and exhaustion of the daily energy budget will influence the flight endurance of budworm females, thus determining where they land after dispersal. This is the first mention of factors that may be involved in the landing process in spruce budworm, other than dropping temperature, precipitation, or sunrise (Régnière, Delisle, et al. 2019). Therefore, larger females of spruce budworm are expected to move the farthest from their birth site during dispersal, a trend generally observed in migratory species with dimorphic (Roff and Fairbairn 2007) and monomorphic wings (Altizer and Davis 2010; Dockx 2007; Hillaert et al. 2018). As budworm adults from areas of severe outbreaks are generally small (Blais 1953; Morris 1963), the longest‐range dispersers should come from areas on the margins of the main outbreak where larval density remains relatively low, and the quantity and quality of host foliage is not yet too degraded. This prediction is in line with some radar observations (Boulanger et al. 2017).

In this study, we investigated the changes in flight performance that were induced by a gradient of temperature and natural variation in barometric pressure and morphological traits. As flight mills allow accurate quantification of the differential values of performance in a gradient of treatment conditions or morphological traits (Naranjo 2019), the shape of the response functions produced in this study should be reliable. Because spruce budworm dispersal occurs at different spatial and temporal scales, our flight performance models could serve as underlying building blocks to develop more realistic complex models of dispersal for this species or improve existing ones (Garcia et al. 2022, 2024; Régnière, Delisle, et al. 2019).

## Author Contributions

**Lucie Royer:** conceptualization (lead), formal analysis (supporting), funding acquisition (supporting), investigation (lead), methodology (lead), visualization (supporting), writing – original draft (lead), writing – review and editing (equal). **Jacques Régnière:** conceptualization (supporting), formal analysis (lead), funding acquisition (lead), visualization (lead), writing – original draft (supporting), writing – review and editing (equal).

## Conflicts of Interest

The authors declare no conflicts of interest.

## Data Availability

The in‐house VBA Excel program to quantify flight performance and data that support the findings of this study are openly available on Canada's Open Data Portal at https://doi.org/10.23687/6231ec26‐b922‐491c‐828b‐3fd485d6d9d7 (Royer and Régnière 2025).

## Appendix 1

**TABLE A1:** Descriptive statistics and normalisation transformation (Box‐Cox exponent or logarithm) of the six morphological variables selected as potential flight performance predictors. The Anderson‐Darling (AD) normality test probability was maximized to select the best normalisation transformation (*y'* = *y* ^*λ*^).

Variable	*N*	Mean	StDev	Minimum	Maximum	Box‐Cox *λ*	AD test
Initial mass (mg)	441	38.80	13.57	11.15	77.98	0.3	0.029
Forewing length (mm)	404	9.81	0.99	6.96	12.55	1	0.384
Total wing area (mm^2^)	402	129.03	26.08	58.34	205.12	0.65	0.129
Wing load (mg/mm^2^)	392	0.29	0.054	0.16	0.48	Log	0.336
Flight muscle ratio	389	0.066	0.008	0.051	0.123	1	< 0.005
Wing aspect ratio	402	3.02	0.18	2.27	3.67	2	0.037

**TABLE A2:** Cross‐correlations between the six morphological variables selected as potential flight performance predictors.

Variable	Initial mass	Forewing length	Total wing area	Wing load	Flight muscle ratio
Forewing length	0.866**				
Total wing area	0.917**	0.959**			
Wing load	0.898**	0.619**	0.666**		
Flight muscle ratio	−0.111*	−0.121*	−0.054	−0.171**	
Wing aspect ratio	−0.191*	0.082	−0.191*	−0.203**	−0.139**

*Probability(*r* = 0) < 0.05, **Probability(*r* = 0) < 0.005.
